# Observer Performance Evaluation of a Deep Learning Model for Multilabel Classification of Active Tuberculosis Lung Zone-Wise Manifestations

**DOI:** 10.7759/cureus.44954

**Published:** 2023-09-09

**Authors:** James Devasia, Hridayanand Goswami, Subitha Lakshminarayanan, Manju Rajaram, Subathra Adithan

**Affiliations:** 1 Preventive Medicine, Jawaharlal Institute of Postgraduate Medical Education and Research, Puducherry, IND; 2 Radiology, Marwari Hospitals, Assam, IND; 3 Pulmonary Medicine, Jawaharlal Institute of Postgraduate Medical Education and Research, Puducherry, IND; 4 Radiodiagnosis, Jawaharlal Institute of Postgraduate Medical Education and Research, Puducherry, IND

**Keywords:** efficientnet, kappa, active tuberculosis, deep learning, observer performance test

## Abstract

Background

Chest X-rays (CXRs) are widely used for cost-effective screening of active pulmonary tuberculosis despite their limitations in sensitivity and specificity when interpreted by clinicians or radiologists. To address this issue, computer-aided detection (CAD) algorithms, particularly deep learning architectures based on convolution, have been developed to automate the analysis of radiography imaging. Deep learning algorithms have shown promise in accurately classifying lung abnormalities using chest X-ray images. In this study, we utilized the EfficientNet B4 model, which was pre-trained on ImageNet with 380x380 input dimensions, using its weights for transfer learning, and was modified with a series of components including global average pooling, batch normalization, dropout, and a classifier with 12 image-wise and 44 segment-wise lung zone evaluation classes using sigmoid activation.

Objectives

Assess the clinical usefulness of our previously created EfficientNet B4 model in identifying lung zone-specific abnormalities related to active tuberculosis through an observer performance test involving a skilled clinician operating in tuberculosis-specific environments.

Methods

The ground truth was established by a radiologist who examined all sample CXRs to identify lung zone-wise abnormalities. An expert clinician working in tuberculosis-specific settings independently reviewed the same CXR with blinded access to the ground truth. Simultaneously, the CXRs were classified using the EfficientNet B4 model. The clinician's assessments were then compared with the model's predictions, and the agreement between the two was measured using the kappa coefficient, evaluating the model's performance in classifying active tuberculosis manifestations across lung zones.

Results

The results show a strong agreement (Kappa ≥0.81) seen for lung zone-wise abnormalities of pneumothorax, mediastinal shift, emphysema, fibrosis, calcifications, pleural effusion, and cavity. Substantial agreement (Kappa = 0.61-0.80) for cavity, mediastinal shift, volume loss, and collapsed lungs. The Kappa score for lung zone-wise abnormalities is moderate (0.41-0.60) for 39% of cases. In image-wise agreement, the EfficientNet B4 model's performance ranges from moderate to almost perfect across categories, while in lung zone-wise agreement, it varies from fair to almost perfect. The results show strong agreement between the EfficientNet B4 model and the human reader in detecting lung zone-wise and image-wise manifestations.

Conclusion

The clinical utility of the EfficientNet B4 models to detect the abnormalities can aid clinicians in primary care settings for screening and triaging tuberculosis where resources are constrained or overburdened.

## Introduction

The global tuberculosis (TB) report highlights the presence of approximately 10.6 million individuals suffering from active cases of tuberculosis. Tragically, TB claims the lives of around 1.4 million individuals annually, within a 95% uncertainty interval (UI) of 1.3 to 1.5 million. Moreover, an additional 187,000 deaths occur due to TB in individuals co-infected with HIV, despite the fact that TB is preventable and curable [1]. Although various screening, triaging, and diagnostic methods have been developed to detect TB, the early identification of pulmonary TB remains a significant challenge in clinical settings where confirmatory tests such as molecular methods, smear microscopy, and culture may be limited or unavailable. Timely detection of active TB is of utmost importance, both for the effective treatment of the individual and for implementing public health interventions aimed at controlling the spread of TB.

Chest X-ray (CXR) is a widely used diagnostic imaging technique in the medical field, serving purposes such as screening, diagnostic evaluations, and monitoring of various thoracic diseases [2,3]. Its primary objective is the early detection and treatment of active pulmonary tuberculosis manifestations. However, CXR is susceptible to reading errors, with low levels of agreement among different observers and even within the same observer, due to limitations in spatial resolution, interference from overlapping anatomical structures, and variations in radiologists' perceptual abilities [3]. However, the World Health Organization (WHO) mandates its use for TB screening [4] and in India's National Tuberculosis Elimination Programme (NTEP), CXR is prioritized for screening, followed by further testing. However, a lack of trained clinicians and radiologists, as well as variability in interpretations, delay diagnosis in high-TB-burden areas. Limited access to tools and systemic gaps hinders prevention efforts in densely populated regions [3,4].

Deep learning (DL) technology has emerged as a potent solution for intricate scientific challenges, with convolutional neural networks (CNNs) showing promise in image analysis due to their extensive parameters gained from data-driven training [5-8]. They have achieved human-like recognition and segmentation in visual tasks [9,10], and advanced machine learning algorithms have excelled in medical image analysis due to large datasets [11]. Earlier studies have highlighted that deep learning systems, trained on such data, match radiologists in diagnosing thoracic diseases [6], retinal images [12], and skin cancers [13]. The artificial intelligence (AI) community is keen on computer-aided diagnosis (CAD) systems in radiology, supporting accurate TB identification using AI methods [14-20]. This aligns with 2021 World Health Organization guidelines endorsing CAD for automated TB screening and triage in individuals aged 15+ due to their substantial representation in reported TB cases [21].

The incorporation of AI algorithms in radiology, specifically via computer-aided detection systems, presents a valuable tool for radiologists in deciphering intricate radiological images. By automating analysis and preliminary screening, these systems enable the prioritization of critical cases and accelerate treatment commencement. Nevertheless, the tangible impact of DL systems in clinical practice remains ambiguous, with limited large-scale clinical assessments. However, the availability of radiologists as a percentage of the overall workforce is displaying a concerning decline across countries. A stark example is India, with its billion-plus population, having fewer than 10,000 radiologists, leading to a strikingly uneven ratio of just one radiologist for every 100,000 individuals. This figure pales in comparison to the US, where the ratio is 1:10,000 [22]. Developing nations like those in Africa experience an even graver scenario, with less than one radiologist per 100,000 people. As populations grow and radiological exams surge, the proportion of qualified radiologists dwindles, causing bottlenecks and delays in crucial medical imaging. Moreover, increased scan volumes coupled with declining radiologist numbers fuel burnout and elevate the risk of interpretation errors.

To address these challenges, implementing a system that automates the quantification task in chest radiographs could alleviate the burden on radiologists. By automating this process, radiologists would have one less aspect to worry about while ensuring that this crucial parameter is not omitted from radiology reports, thereby saving time and improving efficiency. Herein, we evaluated the clinical utility of our previously developed EfficientNet B3 model in detecting lung zone-wise abnormalities associated with active tuberculosis [23]. To achieve this, we conducted an observer performance test with an experienced clinician working in tuberculosis-specific settings. We believe this study represents one of the initial efforts to evaluate the observer performance of lung zone-wise manifestations of active TB. The findings have the potential to expand the application of our model in real-world primary care settings with limited resources.

## Materials and methods

Study design

This study was a cross-sectional design.

Inclusion criteria

Expert radiologists and clinicians with more than five years of experience in reading CXR.

Sampling 

The study's target population comprises experienced radiologists and clinicians affiliated with diverse institutions. We adopted a purposive sampling approach, selecting participants based on their extensive experience interpreting chest X-rays (CXRs) in regions with a high prevalence of tuberculosis. Additionally, we considered their willingness to analyze 100 CXRs, conveniently selected for all lung abnormalities, systematically divided by lung zones. The sample size for this study was established as one expert clinician/radiologist and 100 CXR.

Dataset sources and curation

In this study, we retrospectively gathered chest X-ray images and patient demographic data from the referral register of the National TB Elimination Programme in a specific Designated Microscopy Center (DMC) between 2021 and 2022. The patients included in the study were diagnosed with active tuberculosis based on physician evaluation or microbiological examinations as per program guidelines. To obtain the chest X-ray images, we accessed the Picture Archival and Communication System (PACS), which employs the Health Level Seven International (HL7) integration system to connect with the Health Information System. By using a unique patient identifier, we retrieved the relevant images from PACS, ensuring that the X-ray date was within a week of the date of the sputum examination results. To protect patient privacy, all the CXR images used in the study underwent de-identification procedures, generating a system-generated study identifier and removing any overlay information present in the X-rays. The chest X-ray images were stored in the tag image file format (TIFF) with a color depth of 24 bits per pixel. A digital radiography modality was employed, with a resolution of 1530 x 1896 pixels. All the chest X-rays were captured using a Portable Samsung Retrofit DR System. In total, we collected 100 chest X-ray images from 100 patients who were confirmed to have active TB and were older than 15 years of age.

Ground truth

A single radiologist (HG) rigorously reviewed posteroanterior chest X-rays depicting active tuberculosis using an approved format by the institute research monitoring committee. To ensure reliability, a validation process involved independent assessments by a radiologist (SA) and a pulmonologist (MR) on 30% of the CXR. The results showed strong agreement between HG and the peer assessors. HG's agreement with SA was nearly perfect (Kappa = 0.83, 95% CI: 0.72-0.93), while an agreement with MR was substantial (Kappa = 0.80, 95% CI: 0.71-0.94). 

Deep learning model

In this study, we employed the EfficientNetB4 architecture, a previously documented framework, for its comprehensive specifications regarding design, training approaches, validation methodologies, and outcome evaluations [23]. The architecture capitalizes on the concept of compound scaling to achieve enhanced performance while concurrently optimizing computational efficiency by curbing parameter count and floating-point operations per second (FLOPs). Our training process encompassed pre-trained networks on the ImageNet dataset, configuring the input image dimensions for EfficientNetB4 as 380 x 380. We initialized the network using weights sourced from ImageNet, effectively harnessing transfer learning. Within the foundational model, we substituted the classifier with the subsequent components: (1) a global average pooling layer (GAP), (2) a batch normalization layer (BN), (3) a dropout layer (D), and (4) a classifier layer featuring 12 classes for image-wise analysis and 44 classes for segment-wise evaluation of lung zones, activated by the Sigmoid function. Notably, the classifier layer encompasses 12 abnormality categories in the image context, encompassing 30 abnormalities in bilateral upper, middle, and lower zones, along with 14 distinct abnormalities identified within left and right lung findings.

Study procedure 

In this study, the performance assessment of a multilabel lung manifestation classification model involved a direct comparison with the interpretations made by an expert clinician with extensive experience in reading chest X-rays in high tuberculosis burden settings within Tamil Nadu's Tiruvannamalai district. This clinician, holding a postgraduate degree in chest medicine and boasting over five years of experience analyzing more than 35,000 CXR cases annually, was enlisted after obtaining informed consent. The clinician, blinded to ground-truth information, was presented with 100 chest X-ray images and tasked with documenting abnormalities in a standardized format, segmented by lung zones. Simultaneously, the EfficientB4 model was employed to classify the same set of CXR images. The expert clinician readings were then compared with the model classification to evaluate the model's performance.

Statistical analysis

The Kappa statistic was used to evaluate the agreement between the expert clinician and the deep learning model in classifying lung manifestations in chest X-ray samples. The confidence interval (CI) for the Kappa coefficient was obtained using the Wilson Score method. Statistical analysis was done using the Python 3.7 statistical library (Python Software Foundation, DE, USA, https://python.org), and a P-value of 0.05 (two-tailed) was considered statistically significant.

## Results

Data description

Manifestations of tuberculosis are discernible within CXR through the identification of distinct patterns within the images. The observed abnormalities in the CXR were systematically categorized into parenchymal and pleural segments, collectively presented in Table 1. In all CXR evaluations, pulmonary opacities were consistently observed, with over 50% of cases exhibiting cavity formations and fibrotic lesions. Additionally, approximately 50% of the CXR analyses revealed indications of pleural thickening, while approximately 33% displayed tracheal shifts. Notably, around 25% of the cases indicated a shift towards the right lung.

**Table 1:** Distribution of radiological abnormalities—image-wise and lung zone-wise representation (N = 100). RUZ: right upper zone, RMZ: right mid zone, RLZ: right lower zone, LUZ: left upper zone, LMZ: left mid zone, LLZ: left lower zone, CXR: chest X-ray. All values represented frequency (n) format.

Abnormality in CXR	Image-wise	Right lung			Left lung		
		RUZ	RMZ	RLZ	LUZ	LMZ	LLZ
Parenchymal							
Opacity	100	76	70	57	75	76	51
Fibrosis	57	44	22	7	36	24	8
Cavity	54	37	16	5	17	15	2
Collapsed lung	48	29	9	3	19	9	10
Calcification	8	8	7	4	4	7	4
Pleural							
Thickening	46	35			24		
Pneumo/hydro pneumothorax	5	3			2		
Effusion/loculated collection	5	2			3		
Overall							
Tracheal shift	33	25			8		
Volume loss	27	17			11		
Mediastinal shift	8	5			3		
Emphysema/hyperinflations	8	8			8		

Agreement statistics of image-wise abnormality

**Figure 1:**
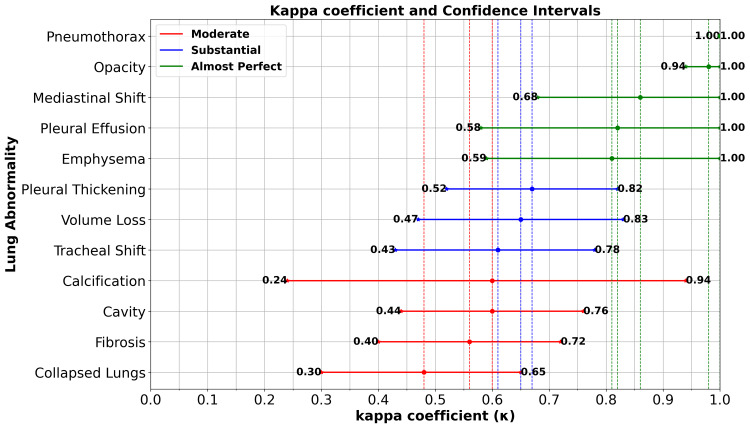
Cohen's Kappa coefficient (κ) and the 95% confidence interval of image-wise abnormalities between EfficientNet B4 and the clinician.

The agreement analysis between the EfficientNet B4 model and the experienced human reader for various abnormalities on chest X-rays (CXR) provides valuable insights into their comparative performance. The findings (Figure 1) reveal that the model demonstrated exceptional agreement (Kappa ≥ 0.81) with the human reader in detecting abnormalities like emphysema, pleural effusion, opacity, and pneumothorax. This indicates that the model has the potential to be a reliable tool for identifying these conditions on CXR images. For abnormalities such as mediastinal shift, cavity, and calcification, the model exhibited substantial agreement (Kappa = 0.61-0.80). This implies that the model can be a valuable aid in detecting these abnormalities. Moderate agreement (Kappa = 0.41-0.60) was observed for collapsed lungs, tracheal shift, fibrosis, volume loss, and pleural thickening. These findings indicate that the model has some ability to detect these abnormalities but may benefit from further refinement to enhance its performance in these areas. It is important to note that despite the model's high agreement in certain abnormalities, it achieved varying levels of agreement across different zones and lung sides.

Agreement statistics of lung zone-wise abnormalities

The findings (Table 2) show that within the substantial agreement group (0.61-0.80), which accounted for approximately 18% of the total classes, 8 classes displayed substantial agreement between the model and the human reader. These included cavity detection in the right middle zone (RMZ) and right upper zone (RUZ) with Kappa of 0.63 and 0.64, respectively, as well as mediastinal shift detection in the right side (R) with a Kappa of 0.79. Additionally, volume loss was observed to fall into this category, with a narrow difference between the right and left lungs. In the almost perfect agreement group (0.81-1.00), representing around 25% of the total classes, 11 classes demonstrated high levels of agreement between the model and the human reader. Notable examples included pneumothorax detection in the right side (R) with a Kappa of 0.85, emphysema detection in the left lung (L) with a Kappa of 0.86, and fibrosis detection in the lower left zone (LLZ) with a Kappa of 0.92. Calcification detection in the left middle zone (LMZ) showed a high agreement with a Kappa of 0.94, while pleural effusion detection in the right side (R) displayed nearly perfect agreement with a Kappa of 0.96. Notably, pneumothorax detection in the left side (L) exhibited perfect agreement with a Kappa of 1.00. Overall, the model's performance varied across the different Kappa categories. While the majority of the classes fell into the moderate to almost perfect agreement groups, fair agreement was observed in certain classes.

**Table 2:** Cohen's Kappa coefficient (κ) and the 95% confidence interval of lung zone-wise abnormality (N = 100). κ: Kappa coefficient, RUZ: right upper zone, RMZ: right mid zone, RLZ: right lower zone, LUZ: left upper zone, LMZ: left mid zone, LLZ: left lower zone, R: right lung, L: left lung. CI: confidence interval. 95% CI for the Kappa coefficient calculated using the Wilson Score method.

Fair (0.21-0.40)		Moderate (0.41-0.60)		Substantial (0.61-0.80)		Almost perfect (0.81-1.00)	
Abnormality	κ (95% CI)	Abnormality	κ (95% CI)	Abnormality	κ (95% CI)	Abnormality	κ (95% CI)
Cavity RLZ	0.26 (0.07–0.90)	Cavity LMZ	0.41 (0.11–0.71)	Cavity RMZ	0.63 (0.41–0.85)	Pneumothorax R	0.85 (0.56–1.00)
Fibrosis LMZ	0.27 (0.02–0.51)	Cavity LUZ	0.43 (0.18–0.69)	Cavity RUZ	0.64 (0.48–0.80)	Mediastinal shift L	0.85 (0.56–1.00)
Fibrosis RLZ	0.33 (0.14–0.81)	Calcification RMZ	0.43 (0.06–0.92)	Collapsed lung LUZ	0.65 (0.44–0.85)	Emphysema L	0.86 (0.68–1.00)
Opacity LMZ	0.36 (0.16–0.56)	Tracheal shift L	0.43 (0.02–0.84)	Volume loss R	0.67 (0.47–0.88)	Fibrosis LLZ	0.92 (0.84–0.99)
Collapsed lung RLZ	0.37 (0.11–0.86)	Opacity RUZ	0.49 (0.28–0.69)	Volume loss L	0.68 (0.41–0.95)	Calcification LMZ	0.94 (0.87–1.00)
Fibrosis RMZ	0.38 (0.13–0.62)	Fibrosis LUZ	0.49 (0.30–0.68)	Emphysema R	0.69 (0.40–0.99)	Calcification LUZ	0.95 (0.89–1.00)
Opacity LUZ	0.40 (0.21–0.58)	Collapsed lung RMZ	0.49 (0.19–0.79)	Collapsed lung LLZ	0.76 (0.52–0.99)	Calcification RLZ	0.95 (0.89–1.00)
Opacity RLZ	0.40 (0.22–0.58)	Opacity RMZ	0.50 (0.32–0.69)	Mediastinal shift R	0.79 (0.50–1.00)	Pleural effusion R	0.96 (0.90–1.00)
		Opacity LLZ	0.50 (0.33–0.67)			Cavity LLZ	0.97 (0.92–1.00)
		Pleural thickening R	0.50 (0.31–0.69)			Calcification LLZ	0.97 (0.92–1.00)
		Calcification RUZ	0.52 (0.12–0.93)			Pneumothorax L	1.00 (1.00–1.00)
		Collapsed lung RUZ	0.53 (0.35–0.07)				
		Fibrosis RUZ	0.56 (0.40–0.73)				
		Pleural effusion L	0.56 (0.06–1.00)				
		Pleural thickening L	0.58 (0.38–0.78)				
		Collapsed lung LMZ	0.59 (0.32–0.86)				
		Tracheal shift R	0.59 (0.40–0.79)				

The classes exhibiting moderate agreement encompassed various abnormalities such as cavitation, calcification, collapsed lungs, fibrosis, and pleural thickening. Some specific examples included cavity detection in the left middle zone (LMZ) with a Kappa of 0.41 and tracheal shift detection in the right side (R) with a Kappa value of 0.59. 

In terms of agreement categories, the findings revealed that 18% of the abnormalities fell into the fair agreement range (0.21-0.40). Within this range, eight out of the 44 classes demonstrated fair agreement. Notable examples included cavity detection in the right lower zone (RLZ) with a Kappa of 0.26 and opacity detection in the RLZ with a Kappa of 0.40. Moving on to the moderate agreement group (0.41-0.60), approximately 39% of the total classes, or 17 classes, were classified in this range.

## Discussion

We assessed the performance of a deep learning-based algorithm in identifying active tuberculosis manifestations in a single chest radiograph image using an observer performance test. The deep learning model demonstrated performance comparable to that of clinicians working in TB-specific settings.

In adults, TB often presents with various findings on chest X-rays, including cavitation and ill-defined opacity in the apical zone and bronchopulmonary segments of the upper and lower lobes. Cavitation, observed in approximately half of the cases, is considered a hallmark of active TB in adults [2,24-26]. While experienced thoracic radiologists can generally differentiate between different active TB manifestations based on a single chest X-ray, determining the activity level solely from one radiographic image can be challenging. Additional information from follow-up radiographs or microbiologic exams is often required to ascertain the activity level accurately. Therefore, the development of an algorithm capable of detecting TB and determining its activity level from the initial screening chest X-ray would be particularly valuable in TB-endemic areas. Such an algorithm could assist in the early identification of active TB cases, enabling timely intervention and treatment initiation.

In a comparative research endeavor undertaken in Vietnam, the evaluative congruence between VinDr-CXR-an EfficientNet-derived system for thoracic disease classification and adept radiologists from Hospital 108 was scrutinized. The investigation uncovered variability in agreement levels between VinDr-CXR and the radiologists across distinct classifications. Regarding the identification of lung opacity, the agreement metric was measured at 0.338 (95% CI: 0.147-0.528). In the instance of pleural effusion, a notably higher agreement was ascertained, reaching 0.921 (95% CI: 0.853-0.989). Correspondingly, the concurrence rate for pleural thickening was 0.337 (95% CI: 0.109-0.565), for pneumothorax it stood at 0.565 (95% CI: 0.125-1.000), and for fibrosis, it was 0.558 (95% CI: 0.386-0.730). By comparison, our experimental undertaking exhibited superior performance when juxtaposed with the VinDr-CXR study, specifically in terms of agreement levels between medical professionals and the deep learning model. For instance, in our investigation, for lung opacity, agreement was notably high at 0.98 (95% CI: 0.94-1.00), for pleural effusion, it registered 0.82 (95% CI: 0.58-1.00), for pleural thickening, it achieved 0.67 (95% CI: 0.52-0.82), for pneumothorax, it attained a perfect score of 1.00 (95% CI: 1.00-1.00), and for fibrosis, it was 0.56 (95% CI: 0.40-0.72). Noteworthy distinctions between the studies encompass the utilization of six major diseases and the inclusion of 22 abnormalities in the VinDr-CXR training, as opposed to our concentrated focus on tuberculosis and the incorporation of 12 image-wise abnormalities and 44 lung zone-wise abnormalities [27]. In a separate investigation, the utilization of Inception V3, a convolutional neural network (CNN) comprised of 159 layers, was employed to discern between endoscopic remission and moderate-to-severe disease. This experiment revealed a near-perfect level of agreement between proficient evaluators and the model (κ = 0.86; 95% CI, 0.85-0.87). A comparable outcome was observed in our own study, wherein the identification of lung abnormalities similarly yielded closely aligned results [28].

In a different study, the performance of radiologists in detecting cardiomegaly was assessed using the Attention U-Net deep learning architecture, which calculated the cardiothoracic ratio (CTR). The study revealed a moderate level of agreement, with a Kappa value of 0.506 [29]. Although our work is not directly comparable, the use of agreement statistics allows for comparison with both human readers and AI models. Another study investigated the difference between human readers and a machine learning model in detecting the differential diagnosis of tuberculous and viral meningitis [30]. The findings indicated a significant difference between the human reader and the machine learning model in their diagnostic capabilities.

This approach will allow for a comprehensive evaluation of the classification model's ability to accurately detect and classify lung manifestations in chest X-ray samples. By comparing the results obtained by expert radiologists with the model's classification results, the study can provide insights into the model's strengths and weaknesses. This can help improve the model and enhance its diagnostic accuracy, which can have significant implications for the early diagnosis and treatment of lung diseases.

A notable strength of this research lies in the utilization of a deep learning model that had been previously published. This study stands as a pioneering effort in its domain, being the first to comprehensively compare the efficacy of a multilabel model and the diagnostic skills of clinicians operating within a specialized tuberculosis context. However, it's important to acknowledge a limitation intrinsic to the methodology. The reliance on a solitary clinician for the interpretation of a diverse array of chest X-rays (CXR) encompassing various abnormal presentations could potentially influence the generalizability of the findings. Moreover, a noticeable research gap exists in the realm of studies dedicated to the classification of lung zone-specific abnormalities in the context of active tuberculosis, along with the accompanying analysis of concordance between human readers and the deep learning (DL) model. This deficit underscores the need for a more comprehensive understanding of DL model performance and its alignment with human experts in the precise identification of abnormalities within distinct lung zones in active tuberculosis cases. The direction for future investigations in this domain becomes evident, emphasizing the necessity to explore the clinical applicability of DL models within radiological settings, as they hold the potential to enhance decision-making processes for clinicians and radiologists alike.

## Conclusions

The outcomes of our study showcased a notable degree of concurrence between the interpretations provided by clinicians and the prognostications generated by the EfficientNet B4-based model. However, it becomes essential to undertake comprehensive clinical evaluations and implementation investigations to grasp the tangible implications and advantages of deep learning systems within the healthcare sphere. These evaluations should be characterized by a stringent assessment of the effectiveness, dependability, and practicality of deep learning algorithms across a spectrum of clinical environments. By doing so, we can effectively expedite the broader assimilation and uptake of deep learning algorithms, thereby harnessing their potential to bolster the early identification of active tuberculosis cases.
